# Recurrent Headache With Diplopia: A Common Presentation of an Uncommon Entity

**DOI:** 10.7759/cureus.56183

**Published:** 2024-03-14

**Authors:** Olusegun J Oluwole, Zeeshan Khan, Ane M Crespo Cuevas, Andrea Lorente Miranda, Vittorio Iantorno

**Affiliations:** 1 Neurology, King's College Hospital, Dubai, ARE; 2 Internal Medicine, King's College Hospital, Dubai, ARE

**Keywords:** painful ophthalmoplegia, ophthalmoplegia, diplopia, migraine, recurrent ophthalmoplegia

## Abstract

Recurrent painful ophthalmoplegic neuropathy (RPON) is a rare headache syndrome, the diagnosis of which can be daunting to those who are not familiar with it. It presents characteristically with recurrent ocular motor weakness and ipsilateral head pain without an underlying etiology and often has unique imaging findings. Even after the successful diagnosis of this entity, there are no published management guidelines. Here, we present the case of a 31-year-old man whom we diagnosed with RPON following two episodes of unilateral headache with ophthalmoplegia over a three-month period and treated successfully with high-dose steroids on both occasions. We highlight the lack of prior migraine history and seeming antecedent viral infection as potential supporting evidence that this condition has a unique pathophysiology different from migraine. We also highlight his dramatic and reproducible response to steroids as additional evidence that steroids are good acute treatment options for this condition. Finally, as our patient lacked the expected cranial nerve imaging abnormalities on head MRI, we suggest that cranial nerve thickening and/or enhancement on MR imaging is not a sine qua non for this diagnosis, contrary to the opinion of some experts.

## Introduction

Recurrent painful ophthalmoplegic neuropathy (RPON) is an uncommon condition with a reported annual incidence of 0.7 per million population [1]. It is characterized by headache and recurrent ocular motor paralysis without identifiable underlying secondary etiologies [2]. In order to diagnose this condition, the International Classification of Headache Disorders, 3rd edition beta version (ICHD3β) requires the presence of recurrent paralysis of one or more of cranial nerves III, IV, and VI associated with ipsilateral headache and exclusion of secondary causes by appropriate imaging of the orbit, parasellar region and posterior fossa [3]. RPON was previously considered a rare subtype of migraine (ophthalmoplegic migraine), but current consensus has reclassified the entity as a painful cranial neuropathy rather than a type of migraine [4,5]. The condition is not familiar to many physicians; hence, it is not surprising that correct diagnosis is often delayed, as was the case of an 11-year-old girl whose diagnosis was made only after four years of recurrent episodes [6]. Even worse is the fact that misdiagnosis can lead to erroneous treatments with potential side effects. Here, we present our case of RPON, highlighting features that are unique in comparison to previously reported cases while equally contributing our treatment experience, as there are currently no consensus guidelines on how to best manage this rare but intriguing condition. We also reflect briefly on the likely pathobiological mechanism underlying this entity, which remains a subject of ongoing debate, especially concerning whether it should be best regarded as a painful cranial neuropathy distinct from migraine or rather a rare migraine subtype. Ethical approval was obtained from the Research and Ethics Committee of King's College Hospital Dubai, and the patient's informed consent was obtained before this case was written up.

## Case presentation

A 31-year-old man presented with a three-week history of progressively worsening headaches for which he had required frequent analgesics. Towards the end of the third week, his headache, which had been holocephalic initially, became more intense and more lateralized towards the right temple as well as the right peri- and retro-orbital areas. At the end of the third week, he started developing double vision and drooping of the right eyelid, which became maximal over the following five days. He denied any obvious redness, tearing, eye discharge, eye swelling, or visual loss. There was mild light sensitivity and subjective feeling of imbalance, but there was no sound sensitivity or prominent nausea. He denied any prior history of recurrent headaches. He had no fever, rash, nasal symptoms, or other systemic symptoms at presentation. Concerning his past medical history, he had been prescribed oral doxycycline and acyclovir seven days before headache onset as treatment for asymptomatic chlamydia and herpes simplex 2 (HSV2) infections detected on polymerase chain reaction (PCR) testing of his urine during a routine check for sexually transmitted infections. There was a family history of migraine in his mother.

**Figure 1:**
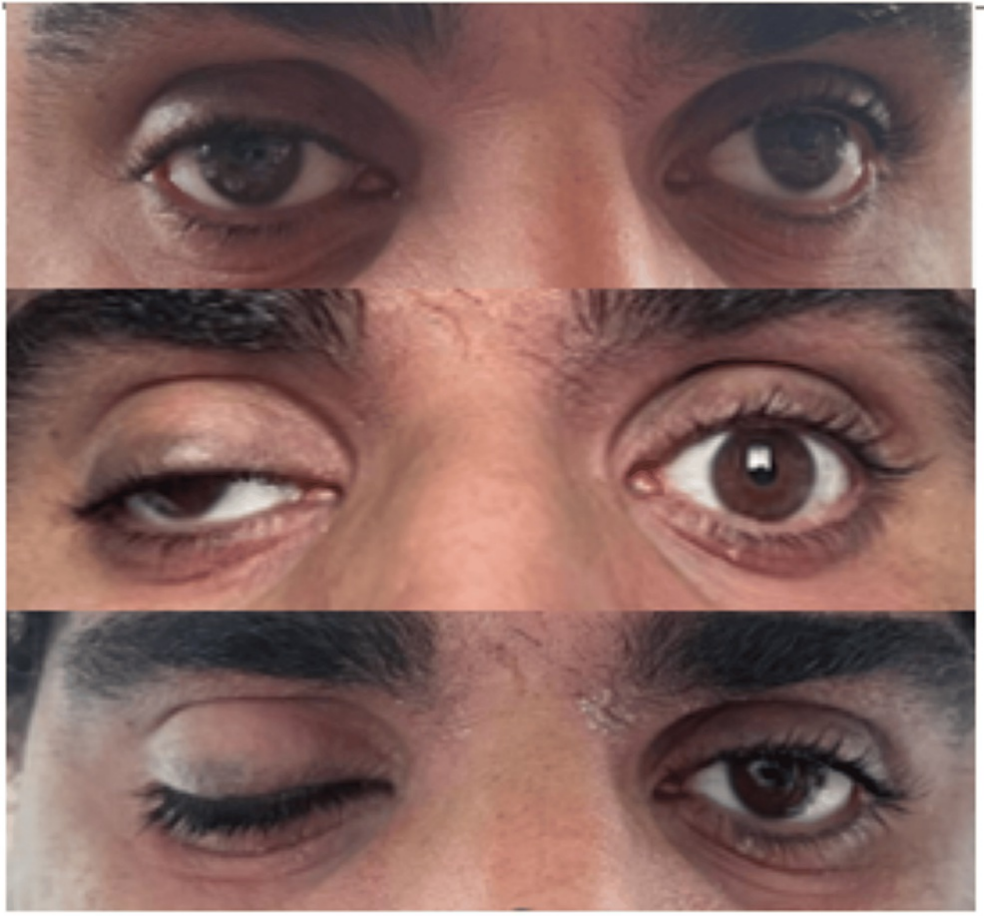
Progression of right cranial nerve (CN) III palsy from first to fifth days The image on top shows the condition on the first day; partial drooping of the right eyelid, concealing the upper margin of the ipsilateral pupil, was observed. The image in the middle demonstrates the condition on the third day; further progression of eyelid drooping with some displacement of the right eye downwards and outwards in primary (central) gaze was observed. The image at the bottom shows the condition on the fifth day; complete drooping of the right eyelid was observed.

On physical examination, he was afebrile and had no rash. Neurological examination revealed complete right oculomotor nerve palsy, as shown in Figure 1.

His pupils were spared. Other ocular motor nerves were normal, as were his optic discs and facial sensation. Visual acuity and color vision were normal, and there were no signs of meningeal irritation. No bruit was heard over his globes.

**Figure 2:**
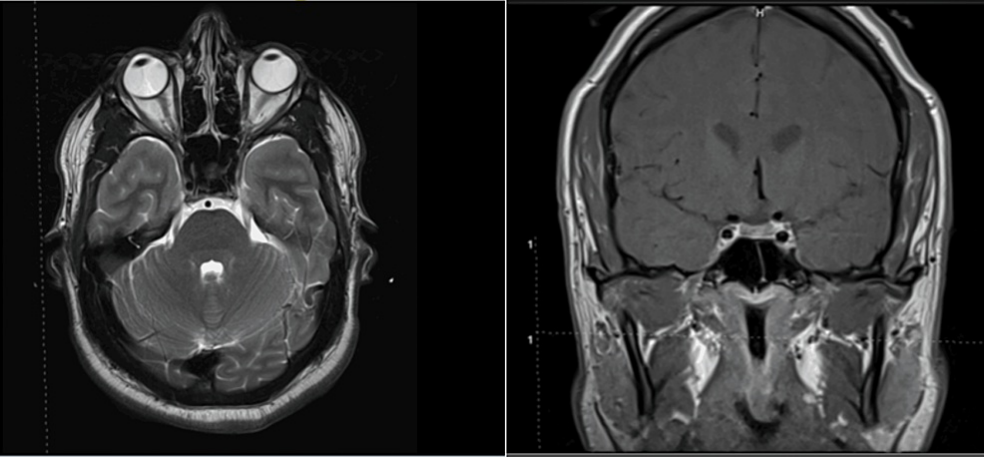
Axial and coronal MR images of the head and orbit showing no abnormalities in the cavernous sinus, orbit, and parasellar regions

A contrast-enhanced MRI of the head did not show any intracranial abnormalities or abnormal enhancement patterns. There were no visible inflammatory lesions nor enhancement patterns within the cisternal portion of the oculomotor nerves. A dedicated contrast-enhanced MRI of the orbit did not show any orbital lesions. Intracranial MRA, digital subtraction angiography (DSA), and magnetic resonance venography (MRV) did not show any aneurysms, fistulae, or venous sinus thrombosis. The patient's head MRI is shown in Figure 2.

A blood draw revealed mild lymphopenia with normal inflammatory markers. Other important blood results are summarized in Table 1.

**Table 1:** Summary of blood tests and CSF results ALT - alanine transaminase; ANCA - anti-neutrophil cytoplasmic antibody; APTT - activated partial thromboplastin time; AST - aspartate transaminase; BUN - blood urea nitrogen; Cr - creatinine; CRP - C-reactive protein; ESR - erythrocyte sedimentation rate; INR - international normalized ratio; i.u/l - international units per liter; PCR - polymerase chain reaction; PT - prothrombin time; TPHA - treponema pallidum hemagglutination assay; VDRL - venereal disease research laboratory; WBC - white blood cells

Tests	Results	Reference values
Blood tests		
Haemoglobin (g/dl)	16.5	13.0-17.0
Hematocrit (%)	51	40-54
WBC count (x10^9^)	8.7	4.5-11.0
Neutrophils (%)	88	42-67
Lymphocytes (%)	10	20-40
CRP (mg/l)	0.9	0.5-4.9
ESR (mm/hr)	10	≤20
BUN (mg/dl)	24	6-24
Cr (mg/dl)	1.05	0.74-1.35
AST (IU/L)	53	6-34
ALT (IU/L)	33	7-40
Fasting plasma glucose (mg/dl)	73	70-100
HbA1c (%)	5.5	4-5.6
HIV 1/2 PCR	negative	negative
Syphilis serology (TPHA)	negative	negative
PT (s)	10.7	11-13.5
APTT (s)	27.8	25-43
INR	1.03	<1.1
P-ANCA	negative	negative
C-ANCA	negative	negative
Angiotensin-converting enzyme (nmol/ml/min)	19	<40
Acetylcholine receptor blocking/binding antibody	negative	negative
CSF tests		
Appearance	clear/colorless	clear
WBC /mm^3^	2 mononuclear cells	0-5
Red cells/mm^3^	0	0
Protein (mg/dl)	20	15-45
Glucose (mg/dl)	49	15-45
Culture	no organisms	no organisms
VDRL	negative	negative
Lyme serology	negative	negative
Oligoclonal bands	negative	negative
Angiotensin-converting enzyme (i.u/l)	0	0.0-2.5

Basic cerebrospinal fluid (CSF) studies, as well as CSF oligoclonal bands, syphilis and Lyme serology, and angiotensin-converting enzyme assay, were similarly unremarkable, as shown in Table 1. Electroencephalography (EEG) showed intermittent generalized slowing in the theta-delta frequency range.

Following mostly normal investigation results, a likely inflammatory oculomotor neuropathy was suspected, and an empirical trial of intravenous (IV) methylprednisolone 1 gr daily was commenced and given for five days. He began to show improvement in all symptoms from the second day of treatment. All symptoms were fully resolved at the end of the third day of treatment with methylprednisolone. He was discharged at the end of the fifth day of treatment to complete a one-week oral prednisolone taper at 60 mg daily. During an outpatient clinic follow-up one week later, he complained of a milder right temporal headache for which topiramate was prescribed and gradually titrated to 50 mg twice daily.

**Figure 3:**
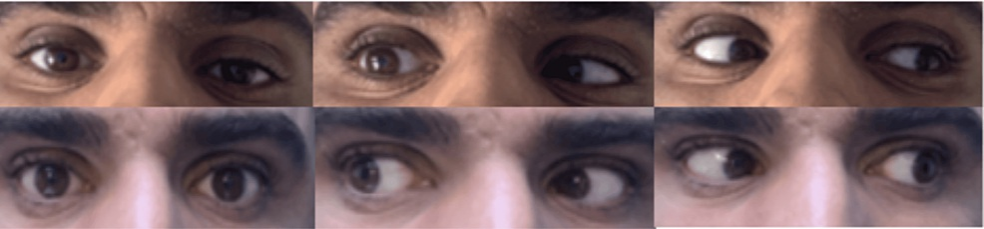
Patient's images at the second presentation, both before and after treatment Images on top show the patient in central gaze, right gaze, and left gaze, respectively, before treatment. Lack of complete abduction of the right eye on right gaze, signifying right lateral recuts weakness, was observed. Images at the bottom show the patient in central gaze, right gaze, and left gaze, respectively, after treatment. Return of complete right eye abduction on right gaze signifying the resolution of lateral rectus palsy after treatment was observed.

He returned three months later with a new attack of right temporal headache associated with double vision, which had begun the previous day. Diplopia was described as horizontal, more prominent upon looking to the right and upon looking at distant objects. The headache lacked migraine features, such as nausea and sensitivity to light and noise. Clinical examination at this time revealed isolated right abducens nerve (cranial nerve, CN VI) palsy, as shown in the top panels of Figure 3.

Repeat contrast-enhanced head MRI, intracranial MRA and MRV, blood draw, and CSF studies were normal, just as they were during the first presentation three months earlier. He was restarted on five doses of IV methylprednisolone 1 gr daily, which resulted in prompt resolution of abducens nerve palsy by the end of the third day of the treatment, as shown in the images at the bottom of Figure 3. The headache also resolved alongside the diplopia. He was discharged home on a five-day course of oral prednisolone 60 mg per day.

Following the second episode of headache with ocular motor paralysis with negative investigations, his diagnosis was revised to recurrent painful ophthalmoplegic neuropathy (RPON). Given his prompt and dramatic response to steroids during both of his attacks, he was given a plan to take oral prednisolone 1 mg/kg for three to five days at the first sign of similar attacks in the future. The frequency of his attacks is currently under study to decide on the need for migraine prophylaxis.

## Discussion

RPON is rare [5], and its presentation may be perplexing to physicians who are unaware of it. Until its reclassification, it was considered a subtype of migraine [3,5]. Despite the reclassification, many experts continue to hold the view that RPON represents a complicated presentation of migraine. For example, one of the largest case series of RPON to date, published from India, described over 60 cases, and all the patients in the series had migraine for variable periods of time before their first presentation of painful ophthalmoplegia [4]. The least duration of migraine before the first presentation of ophthalmoplegia in that large series was three years. Moreover, the attacks of painful ophthalmoplegia occurred during periods of worsening migraine headaches in most of the cases (82%). This led the authors to argue that RPON may be a complication arising from the worsening of migraine attacks. They also proposed pathobiological mechanisms linking intense migraine activity to cranial neuropathy [4].

Our patient had no prior history of migraine, and this might be an indication that RPON can occur in persons without a prior history of migraine. Thus, both conditions may not be necessarily linked. The alternative hypothesis considers RPON an inflammatory cranial neuropathy of post-viral or idiopathic etiology [7]. Our patient's initial white blood cells (WBC) count showed lymphopenia, which could be evidence of antecedent asymptomatic viral infection, which might have initiated a post-infectious inflammatory cranial neuropathy. Depending on which side of the argument one chooses to lean, our patient's persistent headache, which began three weeks before the occurrence of ophthalmoplegia, could be interpreted as due to the new onset of migraine, particularly because of positive family history and the association with dizziness and mild light sensitivity. Furthermore, his EEG at the time had shown intermittent generalized slowing of brain activity, as one could see in migraine. Alternatively, the antecedent prolonged headache without a prior history of headache vulnerability and the context of transient lymphopenia and normal CSF findings may be interpreted as evidence of post-infectious headache from an antecedent viral infection, which eventually culminated in post-infectious cranial neuritis.

The oculomotor nerve is reported to be the most frequently affected nerve in RPON, followed by abducens and trochlear nerves [8]. This has been suggested to be due to the porous nature of the oculomotor nerve's blood-nerve barrier [9]. On the contrary, some authors have reported more frequent involvement of the abducens nerve [4], but these cases appear to be far between. In our case, the oculomotor nerve was affected first and abducens next. Oculomotor nerve involvement takes various patterns. Our case showed complete oculomotor nerve paralysis but without pupillary involvement. However, some patients present with isolated drooping of the eyelid without ocular motor weakness, while others present with pupillary involvement mimicking aneurysmal oculomotor nerve palsy from posterior communicating artery aneurysm [4]. The variety of cranial nerve III presentations makes it imperative to conduct appropriate investigations to exclude more serious conditions. Simultaneous involvement of oculomotor and abducens nerves in a single episode has also been reported [4], though sequential involvement of both nerves in different episodes appears to be more common, as it was in our case.

The criteria put forth by the International Headache Society Classification of Headache Disorders 3rd edition beta version (ICHD 3β) require the exclusion of parasellar and posterior fossa lesions by relevant imaging [3]. The most important differential diagnoses to consider include Tolosa Hunt's syndrome (THS), cavernous sinus thrombosis, superior orbital syndrome, orbital apex syndrome, posterior communicating artery aneurysm, myasthenia gravis, diabetic cranial neuropathy, sarcoidosis, luetic disease, and carotico-cavernous fistula [2,4-6,8]. Each of these entities was carefully considered and excluded via appropriate clinical, laboratory, and radiologic examinations. THS usually causes sensory deficit along V1 and/or V2 territories and is usually associated with a granulomatous inflammatory lesion in the cavernous sinus or orbital apex on head imaging [6]. Our patient had normal investigations besides the lymphopenia on his complete blood count, which normalized upon repeat testing a few weeks later. Normal HbA1c excluded diabetic oculomotor neuropathy, and normal CSF examination helped exclude intracranial infection and active central nervous system (CNS) inflammation. Normal angiotensin-converting enzyme (ACE) level in serum and CSF helped to exclude sarcoidosis, while normal cranial and vascular imaging excluded structural and vascular abnormalities.

Imaging in many cases of RPON has been reported to show thickening of the cavernous portion of the involved ocular motor nerve on unenhanced MR scans and enhancement of the nerve on gadolinium-enhanced scans during the acute phase in most cases [6]. Some believe this is a pointer to the inflammatory nature of this entity. This finding may, however, be absent in up to 25% of cases [10]. Our patient did not show thickening or enhancement of the oculomotor and abducens nerves during either of his presentations. Therefore, the absence of nerve thickening or enhancement should not deter the diagnosis.

To date, no guidelines have been published on how to manage RPON best. This might have to do with the rarity of this entity. As a result, treatment guides may only be gleaned from published case reports and series. Although steroids do not seem to work in all patients [3], there are several case reports and series indicating that steroids may help shorten the time to recovery during an acute attack [9]. Our patient had a dramatic response to steroid treatment during both of his presentations, and we capitalized on his steroid responsiveness when formulating an acute treatment plan for his future attacks. Considering the mounting evidence supporting the efficacy of steroids during acute attacks, it may be a reasonable first choice to consider for acute treatment. Admittedly, some authors have reported a lack of response to steroids [5]. Nonetheless, some of these authors admit to partial response, such as resolution of ophthalmoparesis without pain relief. One such author reported using pregabalin to successfully treat the residual pain after ophthalmoparesis was treated with steroids [2]. Non-steroidal anti-inflammatory drugs (NSAIDs) and β-blocker eyedrops are other agents that have been reported to help in the acute phase [8,11].

Depending on the frequency of attacks, some authors prescribe prophylactic treatments to reduce the number of attacks. Variable levels of success have been reported with the use of topiramate, valproate, and calcium channel blockers, all of which are migraine treatments [2,5,6,8]. Some authors argue that successful prevention of further attacks with these agents suggests shared pathophysiology mechanisms between migraine and RPON [4]. Our patient was prescribed topiramate for a short time after his first attack to help control his residual headaches, but he stopped it as soon as he felt the headaches had improved. We need further follow-up to understand his future frequency of attacks and decide on the need for preventative therapy. Even though the ocular motor deficits in RPON are usually reversible, it is recognized that the deficits may become permanent and irreversible after several years of recurrent attacks [10]; hence, there is a need for physicians to recognize this condition and make appropriate and timely interventions.

## Conclusions

RPON is an uncommon neurological condition that presents with headaches and recurrent ophthalmoplegia. Our case shows features that support both two opposing views about the underlying pathobiology of this condition. The dramatic and sustained response to steroids in our case adds to existing evidence that steroids work in the acute phase, and we suggest that steroids should be considered as first-line options for acute treatment.
